# Effects of a Cardiac Telerehabilitation Program on the Quality of Life and Functional Independence of Patients With Coronary Artery Bypass Graft: A Non-randomized Controlled Trial

**DOI:** 10.7759/cureus.78548

**Published:** 2025-02-05

**Authors:** Satani Kalpesh, Neha Mukkamala, Palani Kumar, P R Jha

**Affiliations:** 1 Cardiorespiratory Physiotherapy, College of Physiotherapy, Sumandeep Vidyapeeth Deemed to be University, Vadodara, IND; 2 Musculoskeletal Physiotherapy, College of Physiotherapy, Sumandeep Vidyapeeth Deemed to be University, Vadodara, IND; 3 Neurophysiotherapy, College of Physiotherapy, Sumandeep Vidyapeeth Deemed to be University, Vadodara, IND; 4 Department of Medicine, Smt. B.K. Shah Medical Institute and Research Centre, Sumandeep Vidyapeeth Deemed to be University, Vadodara, IND

**Keywords:** activities of daily living (adls), cardiac telerehabilitation, coronary artery bypass graft (cabg), exercise program, functional status, quality of life (qol)

## Abstract

Background: The most common surgical approach for the treatment of coronary artery disease is coronary artery bypass graft (CABG), and it helps to lower mortality and morbidity. Cardiac rehabilitation (CR) is a systematic approach for improving patients' physical and functional abilities. It also helps promote independence and reduce the rate of hospital readmissions. Following surgery, there is a relatively low referral rate for CR, and compliance is much more of an issue. Increasing involvement in CR can be achieved by cardiac telerehabilitation. Thus, this study aims to evaluate how cardiac telerehabilitation affects functional independence and quality of life in patients with CABG.

Methods: A non-randomized controlled trial was conducted in a multispecialty hospital for four years in which a total of 49 consecutive CABG patients were recruited and divided into a cardiac telerehabilitation (25 patients, including 12 males and 13 females) and home exercise group (24 patients, including eight males and 16 females) through purposive sampling based on their ability to use audio-visual aid and participate in cardiac telerehabilitation. Functional independence was assessed with the Functional Independence Measure (FIM) scale and quality of life was measured with the Short Form-36 (SF-36) scale at discharge, one month, and three months post discharge.

Results: The mean FIM score of group A was 107.40 (4.72) and group B was 105.92 (2.60), whereas the mean SF-36 score of group A was 35.61 (6.36) and group B was 31.59 (5.18). At three months post discharge, patients in the cardiac telerehabilitation group showed statistically significant improvement in functional status (p = 0.033 at one month and p = 0.019 at three months post cardiac telerehabilitation) and quality of life (p = 0.001 at three months post cardiac telerehabilitation).

Conclusion: Cardiac telerehabilitation is beneficial for patients with CABG, improving their daily living and quality of life if they are unable to attend center-based CR.

## Introduction

Cardiovascular diseases (CVDs) are one of the common non-communicable diseases, a leading cause of morbidity and mortality that is associated with high healthcare costs. Indians are affected with CVDs at least a few years earlier and during the most productive middle age, in contrast to those with Western ancestry. For instance, India has twice the rate of deaths from CVDs before the age of 70 years compared to the West [1]. The results of the Global Burden of Disease study state the age-standardized CVD death rate of 272 per 100,000 populations in India, which is much higher than the global average of 235. The most common surgical approach for the treatment of coronary artery disease (CAD) is coronary artery bypass graft (CABG). It can lower premature mortality in high-risk patients and is done to eliminate CAD symptoms [2].

Immediate decline in physical function is common after CABG, and the reason for the decline could be linked to activity restrictions following surgical procedures to guard incision and anxiety about activity aggravating symptoms of CAD. Following the decline in function after CABG, progressive involvement in household physical activities is important to facilitate an early return to daily activities, including social, vocational, and recreational activities. Numerous qualitative and descriptive studies on CABG patients concluded that the patient's daily function is limited by several surgery-specific characteristics [3]. While most CABG patients can execute basic self-care tasks, they struggle with instrumental activities of daily living (ADLs), which makes them more reliant on family members and reduces their health-related quality of life. However, as patients gain confidence and the length of time after surgery rises, the majority of these ADLs get better [4-6].

An evidence-based interdisciplinary program called cardiac rehabilitation (CR) has been shown to increase independence and quality of life while lowering morbidity, mortality, and the rate of hospital readmissions. As a Class IA recommendation in the 2021 American College of Cardiology (ACC) and American Heart Association (AHA) guidelines, CR includes guided physical activity, health education, risk factor modification, stress management, etc. [7]. Participation in a regular tailor-made structured program of aerobic exercise during the first three months following CABG has been shown to restore physical activities and function and offset any peri-operative deconditioning by improving aerobic capacity and improving lean muscle mass. Any improvement in physical function is significant as it improves the person's capacity to participate in self-help activities [8]. In India, CR involvement is still quite low despite these strong recommendations. Referral rates for CR range from 9% to 39% globally. However, only 6% to 59% of patients who are referred take part in this program. According to studies, the low rate of referral is due to lack of referral by primary surgeons themselves, presence of comorbidities, self-belief in perceived benefits of CR, transportation issues, family and social support, and absenteeism [9,10].

Cardiac telerehabilitation can significantly improve referral rates for CR programs by overcoming geographical barriers, increasing accessibility, and providing flexibility for patients who might otherwise be hesitant to participate in traditional, in-person programs. With the use of portable, remote communication and monitoring equipment, cardiac telerehabilitation can be carried out at home with convenience. It offers many advantages, such as cost-effectiveness, reduced absenteeism from work, a lower re-hospitalization rate, and more, and it can be used in place of center-based CR. Brouwers et al. (2020) state that CR can be crucial in enhancing functional independence, functional capacity, upper extremity function, and quality of life [11].

There is not much research examining how continuous monitoring of an exercise program through cardiac telerehabilitation affects functional independence and quality of life in patients with CABG over time in the Indian rural community. Thus, the purpose of this study was to evaluate the effectiveness of cardiac telerehabilitation on functional independence and quality of life in patients with CABG.

## Materials and methods

A non-randomized controlled trial was conducted at Matsama Heart Center of Dhiraj Hospital of the Vadodara district of Gujarat state of India from 2018 to 2023 in which a total of 49 consecutive CABG patients participated. This research followed the CONSORT (Consolidated Standards of Reporting Trials) guidelines, was approved by the Sumandeep Vidyapeeth Institutional Ethics Committee (SVIEC) (approval number: SVIEC/on/Phys/PhD/12016), and was registered with Clinical Trial Registry India (CTRI) with registration number CTRI/2018/05/013575.

Every consecutive CABG patient from the cardiac surgical intensive care unit (CSICU), referred for physiotherapy, who underwent in-center cardiac rehabilitation during their ICU stay, and had a normal recovery without any postoperative complications like atelectasis, pneumonia, hypoxemia, infection of the surgical site, etc. was recruited in the study after getting their written consent. Both male and female patients who could understand the local language were included in the study; however, patients who experienced postoperative complications during their hospital stay, had cognitive impairments that made it difficult for them to understand the research questionnaires, or had a history of neurological, musculoskeletal, or pulmonary issues before surgery that interfered with their ability to perform ADLs were excluded from the study.

Following eligibility screening, 59 patients (61.61 ± 6.97) ranging in age from 41 to 74 years were enrolled and split into two groups according to their proficiency with technology, such as video calls. Group A, or the continuous monitoring group, consisted of 29 patients who had the facility to make video calls using the WhatsApp application (Meta, Menlo Park, CA), while group B, or the home program group, consisted of 30 patients who lacked this capability and were not very tech-savvy. The same 12-week aerobic and strengthening exercise routine, known as the "Rehabilitation home program," was distributed as a handout to both groups. The rehabilitation home program consisted of an aerobic and strengthening exercise protocol developed as per the American College of Sports Medicine’s (ACSM) FITT (frequency, intensity, time, and type) principle. Each patient (of both groups) was guided individually before beginning the aerobic and strengthening exercise protocol, including measurement of their heart rate and physical exertion.

Patients of group A performed aerobic and strengthening exercises under supervision through cardiac telerehabilitation (video call) for a minimum of twice a week, and on the other days, they were asked to continue exercises. In addition to being urged to keep their daily work diary, group B patients were instructed to follow the exercise guidelines (minimum of twice a week) and to keep exercising and being active. The data of 25 patients in group A (12 males and 13 females) and 24 patients in group B (eight males and 16 females) were examined after 12 weeks due to dropout.

Patients in both groups had their baseline data assessed at the time of discharge from the hospital. The independence in performing ADLs was assessed with the Functional Independence Measure Scale (FIMS) [12], and the quality of life was evaluated using the Short Form-36 (SF-36) scale [13]. Following discharge, these outcome measures were re-evaluated at one-month and three-month intervals, respectively. When patients (of both groups) came in for a follow-up at the end of the first month, they were asked about their progress with the exercises and provided any personal guidance they needed to continue.

The Shapiro-Wilk test was used to assess for normality in the baseline values. The mean differences of the variables, such as the FIMS score and other SF-36 score components, between the two groups were compared using the independent t-test. The significance level was set at 5% in this study.

## Results

**Figure 1:**
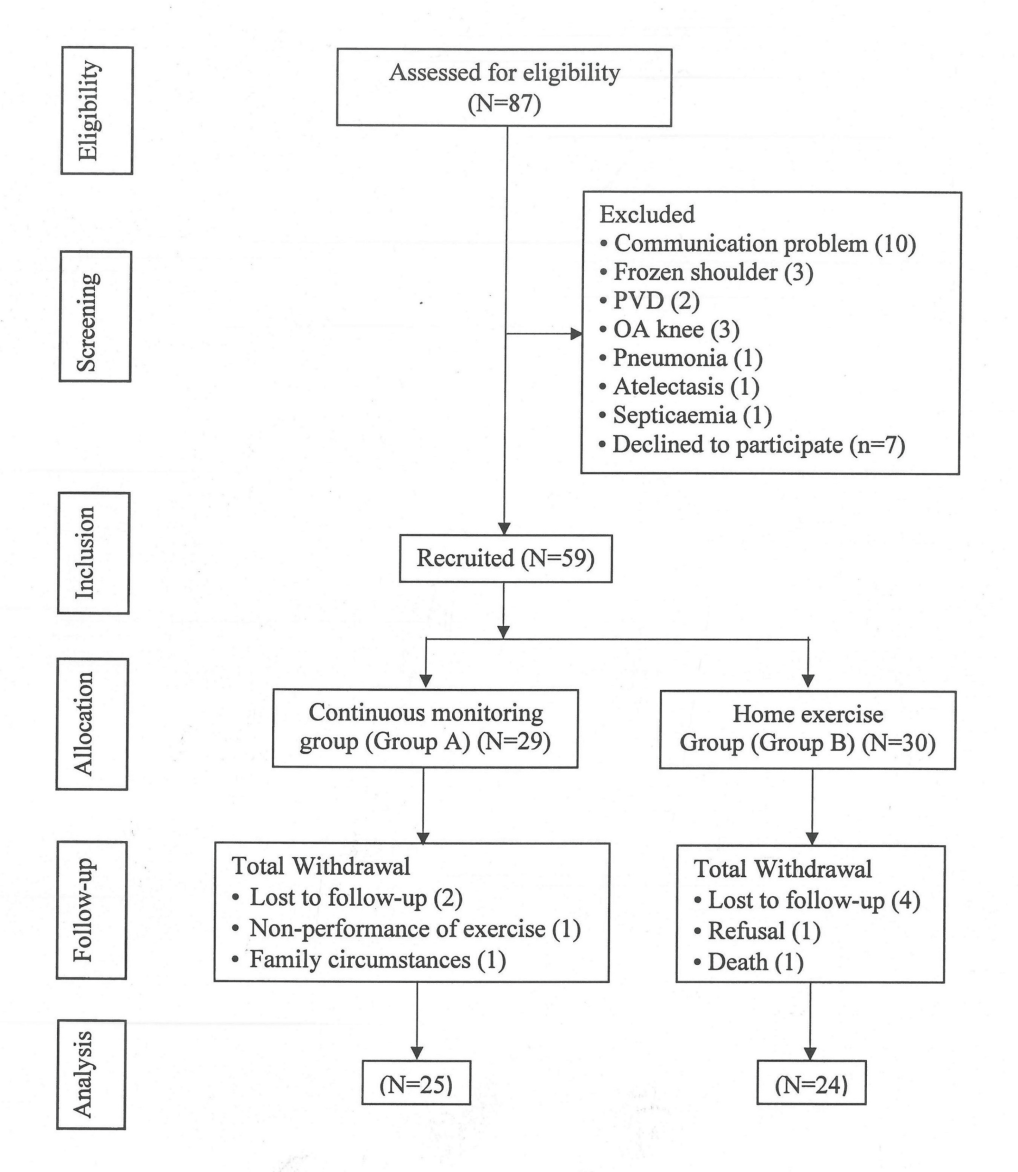
Study flow chart. PVD: peripheral vascular disease; OA: osteoarthritis.

In terms of clinical, surgical, and demographic characteristics, the groups were identical. Table 1 provides a summary of the baseline clinical and demographic data for patients in both groups. There was no significant difference between groups regarding age, gender, history of smoking, left ventricle ejection fraction (LVEF), and number of involved coronary arteries. Similarly, anesthesia and surgical parameters also did not significantly differ between the groups. During the recruitment period, 87 CABG patients were screened for eligibility (Figure 1). Of these, 59 patients were recruited after meeting the requirements for inclusion. Following CABG, all groups got the same treatment according to standard guidelines throughout their initial postoperative hospital stay. Group A consisted of 29 patients who were engaged in cardiac telerehabilitation, whereas group B consisted of 30 patients who were enrolled in a home exercise program. Nevertheless, data from 49 individuals were finally examined after dropout following the three-month follow-ups (Figure 1).

**Table 1:** Baseline demographic and clinical patient characteristics. BMI: body mass index; FIMS: Functional Independence Measure Scale; 6MWT: six-minute walk test; SF-36: Short Form-36.

Variable	Group A (N = 25)	Group B (N = 24)	p-value
Age (years)	61.64 ± 6.14	61.58 ± 7.89	0.978
Gender			
Male	12 (48%)	8 (33.3%)	0.296
Female	13 (52%)	16 (66.7%)	
BMI (kg/m^2^)	24.30 ± 2.10	25.84 ± 2.54	0.025
History of smoking	8 (32%)	8 (33.3%)	0.921
Medical history			
Diabetes mellitus	11 (44.0%)	11 (45.83%)	
Hypertension	15 (60.0%)	11 (45.83%)	
Hypercholesterolemia	4 (16.0%)	8 (33.33%)	
Hyperthyroidism	1 (4.0%)	3 (12.5%)	
Ejection fraction (%)	42.15 ± 5.42	43.5 ± 6.99	0.443
Number of affected vessels			
Single vessel	0 (0.0%)	3 (12.5%)	0.142
Double vessel	8 (32.0%)	9 (37.5%)	
Triple vessel	17 (68.0%)	12 (50.0%)	
FIMS	107.40 ± 4.72	105.92 ± 2.60	0.183
6MWT distance (meters)	208.28 ± 49.42	184.25 ± 44.82	0.082
Hand grip strength (kg force)			
Dominant	24.96 ± 4.84	24.42 ± 4.07	0.674
Non-dominant	22.72 ± 7.59	18.04 ± 5.02	0.015
Quadriceps strength (kg)			
Dominant	2.46 ± 0.78	2.37 ± 0.64	0.683
Non-dominant	2.16 ± 0.75	1.93 ± 0.66	0.282
SF-36 score			
Physical	33.94 ± 8.44	28.64 ± 7.32	0.024
Mental	37.29 ± 8.41	34.54 ± 6.45	0.207
Total	35.61 ± 6.36	31.59 ± 5.18	0.020

**Figure 2:**
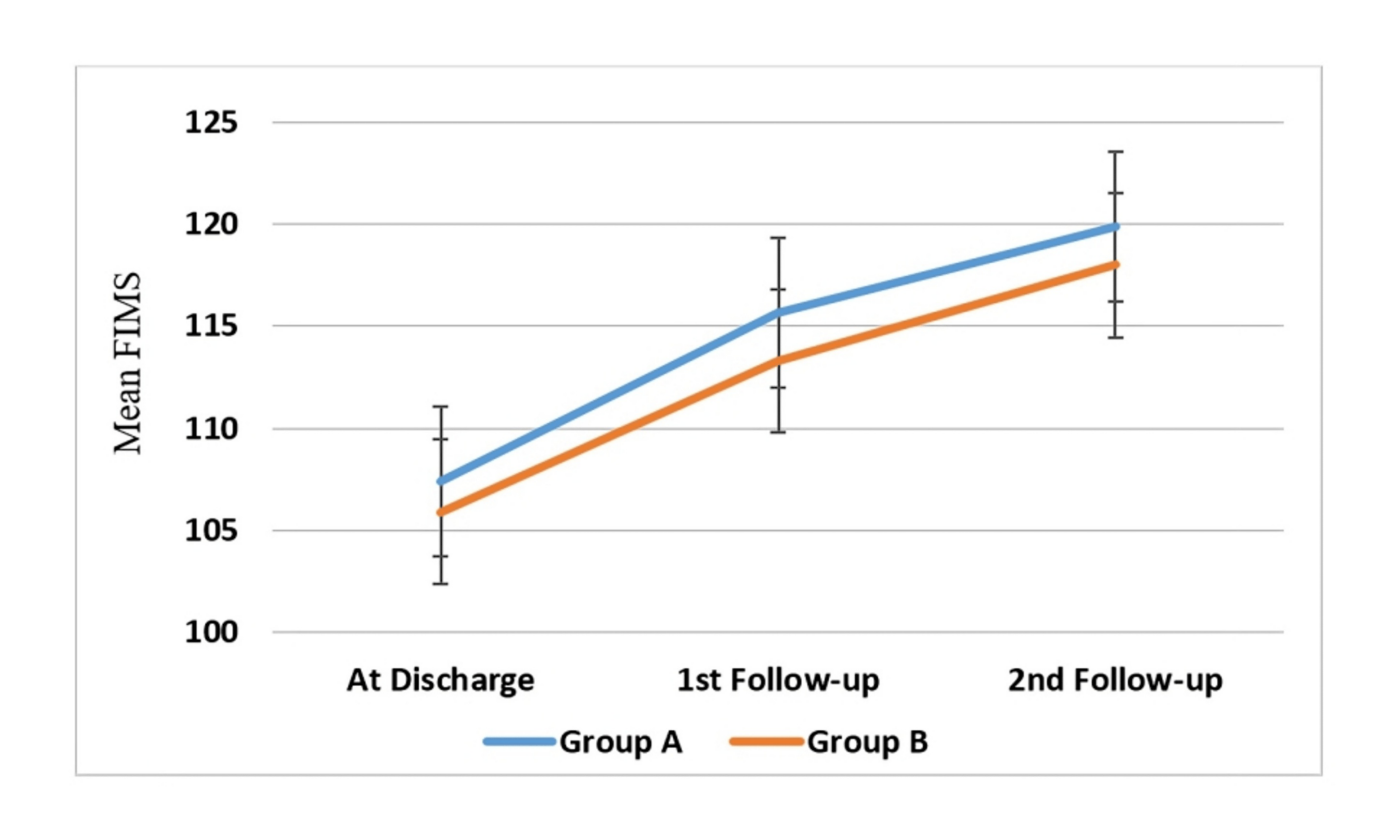
Functional independence at discharge, at one-month, and at three-month follow-up after CABG compared between the cardiac telerehabilitation (group A) and home program group (group B). FIMS: Functional Independence Measure Scale; CABG: coronary artery bypass graft.

Table 2 and Figure 2 illustrate the functional independence of both groups at different post-CABG periods. At discharge, there was no statistically significant difference in the groups' functional independence as determined by FIMS (p = 0.183). Functional independence from discharge to follow-ups significantly improved in both groups. However, at the 1st follow-up (p = 0.033) and the 2nd follow-up (p = 0.019) at a 95% confidence interval, the functional independence of the patients in the cardiac telerehabilitation group was significantly higher than that of the patients in the home program group.

**Table 2:** Comparison of FIMS between groups using independent t-test. FIMS: Functional Independence Measure Scale.

FIMS	Group	N	Mean	SD	Mean diff.	t-value	p-value
At discharge	Group A	25	107.40	4.72	1.483	1.353	0.183
	Group B	24	105.92	2.60			
1^st^ follow-up	Group A	25	115.68	3.76	2.388	0.033	0.033
	Group B	24	113.29	3.85			
2^nd^ follow-up	Group A	25	119.88	2.47	1.880	0.019	0.019
	Group B	24	118.00	2.94			

**Figure 3:**
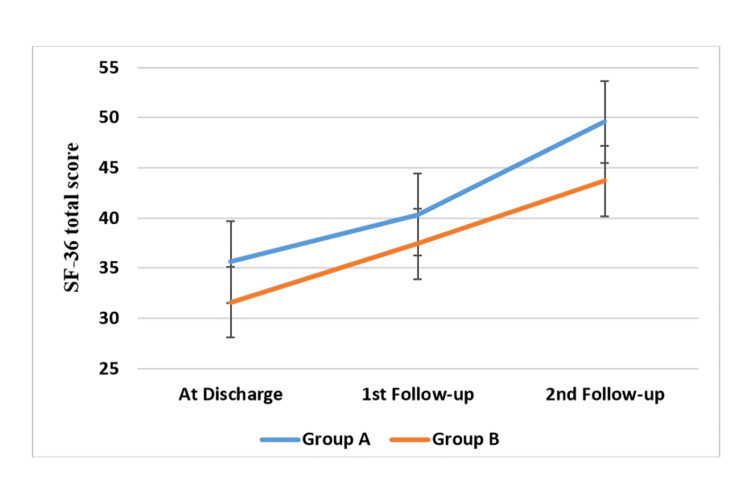
Quality of life (total SF-36 score) at discharge, at one-month, and at three-month follow-up after CABG compared between the cardiac telerehabilitation (group A) and home program group (group B). SF-36: Short Form-36; CABG: coronary artery bypass graft.

Table 3 and Figure 3 show the physical and mental components of quality of life as assessed by the SF-36 at discharge, 1st follow-up, and the 2nd follow-up following CABG in both groups. From discharge to the first and second follow-ups, quality of life significantly improved in both groups. At three months after CABG, however, the cardiac telerehabilitation group showed a significant improvement in both the mental and physical components of quality of life than the control group (p = 0.001 - total; p = 0.002 - physical; and p = 0.025 - mental component).

**Table 3:** Comparison of SF-36 scores (including physical and mental components) between groups using independent t-test. SF-36: Short Form-36.

SF-36		Group	N	Mean	SD	Mean diff.	t-value	p-value
At discharge	Physical	Group A	25	33.94	8.44	5.29	2.340	0.024
		Group B	24	28.64	7.32			
	Mental	Group A	25	37.29	8.41	2.75	1.280	0.207
		Group B	24	34.54	6.45			
	Total	Group A	25	35.61	6.36	4.02	2.418	0.020
		Group B	24	31.59	5.18			
At 1st follow-up	Physical	Group A	25	37.24	9.00	3.59	1.625	0.111
		Group B	24	33.64	6.17			
	Mental	Group A	25	43.46	6.97	2.26	1.137	0.261
		Group B	24	41.20	6.94			
	Total	Group A	25	40.35	5.59	2.92	1.934	0.059
		Group B	24	37.42	4.97			
At 2nd follow-up	Physical	Group A	25	46.38	7.50	6.14	3.252	0.002
		Group B	24	40.23	5.53			
	Mental	Group A	25	52.08	6.99	4.91	2.322	0.025
		Group B	24	47.16	7.82			
	Total	Group A	25	49.59	5.64	5.88	3.697	0.001
		Group B	24	43.70	5.50			

## Discussion

To the best of our knowledge, this is the first study of its kind conducted in India to assess the impact of cardiac telerehabilitation on the functional independence and quality of life of patients undergoing CABG. The findings of this study demonstrated that the cardiac telerehabilitation program combined with continuous monitoring, comprising 12 weeks of aerobic and strengthening exercise training, is associated with significant improvements in the functional independence and health-related quality of life (HRQoL) of patients with CABG.

In a study of functional status and correlates after CABG, DiMattio and Tulman found that women's functional status significantly improved over six weeks [14]. During the study period, the majority of women reported an improvement in their overall functional status, and they were most likely to engage in personal care and low-to-moderate-level household chores. Patients in the cardiac telerehabilitation group in the current study also demonstrated an improvement in their functional status because, following initial sternal precautions, they engaged in instrumental and basic ADLs, increasing their functional independence and decreasing their reliance on family members [15].

Izawa et al. examined how an eight-week CR program affected the physiological outcomes and HRQoL of patients who had suffered an acute myocardial infarction, and concluded that eight weeks of exercise training have particular effects on improvement in HRQoL and physiological outcomes in Japanese patients [16].

Outpatient CR was found to have an impact on HRQoL in patients who had aortic coronary bypass surgery, according to Hsu et al. [17]. Following rehabilitation, these patients' physical functioning, physical role, body discomfort, and social function all showed significant improvements. Karapolat et al. examined the effects of home-based exercise versus hospital-supervised exercise on psychological symptoms, functional capacity, and quality of life in patients following orthotropic heart transplantation. They found that the hospital-based exercise group had significantly improved maximal oxygen consumption and the majority of SF-36 subgroups. These results are supported by the notable improvement in HRQoL we noted in CABG patients [18]. Therefore, the 12-week aerobic and strengthening exercise program through cardiac telerehabilitation improves functional independence and HRQoL, which is very important for patient survival.

The findings of this study have significant implications for clinical practice and healthcare systems. Cardiac telerehabilitation has the potential to improve patient outcomes by increasing accessibility, personalizing care, and reducing costs. For healthcare systems, it offers a scalable and resource-efficient solution to manage CR, particularly in underprivileged areas. Future research should focus on long-term outcomes and the development of innovative technologies to enhance the effectiveness of cardiac telerehabilitation programs. Overall, the adoption of cardiac telerehabilitation can contribute to reducing the global burden of CVDs and promoting health equity.

The current research had several limitations. It comprised a limited number of participants who were not assigned at random to the cardiac telerehabilitation and home program group. Patients were split into groups based on their ability to use video calls, i.e., cardiac telerehabilitation, which might cause selection bias. It was a project conducted at a single center, and the findings might not be applicable to a broader population.

## Conclusions

In conclusion, the results of the study indicate that regular participation in 12 weeks of cardiac telerehabilitation in the form of aerobic and strengthening exercises leads to significant improvement in the quality of life and function independence of patients with CABG. Continuous monitoring of the rehabilitation program will ensure safety during the performance of exercises and will increase the confidence level of patients who need to carry out ADLs during the early rehabilitation phase post surgery.
